# Performance and sex difference in ultra-triathlon performance from Ironman to Double Deca Iron ultra-triathlon between 1978 and 2013

**DOI:** 10.1186/2193-1801-3-219

**Published:** 2014-05-01

**Authors:** Christoph A Rüst, Thomas Rosemann, Beat Knechtle

**Affiliations:** Institute of General Practice and Health Services Research, University of Zurich, Zurich, Switzerland; Gesundheitszentrum St. Gallen, Vadianstrasse 26, St. Gallen, 9001 Switzerland

**Keywords:** Woman, Man, Swimming, Cycling, Running

## Abstract

It was assumed that women would be able to outperform men in ultra-marathon running. The present study investigated the sex difference in performance for all ultra-triathlon distances from the Ironman distance (*i.e.* 3.8 km swimming, 180 km cycling and 42 km running) in the ‘Ironman Hawaii’ to the Double Deca Iron ultra-triathlon distance (*i.e.* 76 km swimming, 3,600 km cycling and 840 km running) between 1978 and 2013. The changes in performance and in the sex difference in performance for the annual three fastest finishers were analysed using linear, non-linear and multi-variate regression analyses from 46,123 athletes (*i.e.* 9,802 women and 46,123 men). Women accounted for 11.9 ± 5.8% of the total field and their percentage was highest in ‘Ironman Hawaii’ (22.1%) and lowest in Deca Iron ultra-triathlon (6.5%). In ‘Ironman Hawaii’, the sex difference decreased non-linearly in swimming, cycling, running and overall race time. In Double Iron ultra-triathlon, the sex difference increased non-linearly in overall race time. In Triple Iron ultra-triathlon, the sex difference increased non-linearly in cycling and overall race time but linearly in running. For the three fastest finishers ever, the sex difference in performance showed no change with increasing race distance with the exception for the swimming split where the sex difference increased with increasing race distance (r^2^ = 0.93, *P* = 0.001). The sex differences for the three fastest finishers ever for swimming, cycling, running and overall race times for all distances from Ironman to Deca Iron ultra-triathlon were 27.0 ± 17.8%, 24.3 ± 9.9%, 24.5 ± 11.0%, and 24.0 ± 6.7%, respectively. To summarize, these findings showed that women reduced the sex difference in the shorter ultra-triathlon distances (*i.e.* Ironman distance) but extended the sex difference in longer distances (*i.e.* Double and Triple Iron ultra-triathlon). It seems very unlikely that women will ever outperform men in ultra-triathlons from Ironman to Double Iron ultra-triathlon.

## Background

A few years ago, Beneke et al. (2005) argued that women would be able to outperform men in ultra-marathon running. They reported that men were dominating the 216 km ‘Badwater’ ultra-marathon held at temperatures up to 55°C during the 1980s and 1990s. However, in 2002 and 2003, a woman was able to outpace the fastest man by ~4.5 h and ~0.5 h, respectively (http://www.badwater.com). Furthermore, since 2002, up to three women finished within the top five overall, although more men than women competed in the ‘Badwater’ (da Fonseca-Engelhardt et al. 2013). A similar finding of a female runner exceeding men’s ultra-marathon performance has been reported for a 45-years old woman winning the ‘Deutschlandlauf’, a multi-stage ultra-marathon covering a total distance of 1,200 km from the North to the South of Germany to be completed within 17 days (Knechtle et al. 2008). The woman finished the race in an overall race time of 124 h 40 min, whereas the first man finished about 8 h behind her in 132 h 44 min.

The assumption that women might outrun men in ultra-marathon running was supported by the comparison of female and male performance in different running distances. Hoffman (2008) showed that women and men who were matched for 50 km trail running performance also similarly performed in trail runs of 80 km and 161 km race distances. Physiological assumptions regarding why women could outrun men were differences in the ability to run aerobically at a higher percentage of maximal oxygen uptake (Riddell et al. 2003), differences in the use of ingested and stored glycogen (Riddell et al. 2003) and differences in lipid metabolism (Perreault et al. 2004). During long-duration exercise at a moderate intensity, women showed a higher lipid utilization and a lower carbohydrate and protein metabolism compared to equally trained and nourished men (Tarnopolsky et al. 1990). In addition, women used more fat stored in the legs compared to men (Roepstorff et al. 2002). Coast et al. (2004) compared by the end of 2002 the world best running performances from 100 m to 200 km. Men were on average ~12.4% faster than women. There was a significant slope to the speed difference across distances where longer distances were associated with greater sex differences (Coast et al. 2004). Therefore, the distance for a woman to outperform a man should be most probably longer than 200 km as it has been found in the 216 km ‘Badwater’ and the 1,200 km ‘Deutschlandlauf’. However, other reasons such as participation trends and motivational trends might limit women’s ultra-endurance performance. Generally, men are over-represented in sports (Deaner 2013) and the better male performance is most probably due to men’s greater training motivation (Deaner 2013). In contrast, women in ultra-endurance are rather task-oriented and internally motivated (Krouse et al. 2011).

In ultra-endurance, long-distance triathlon is an interesting model investigating the sex difference in ultra-endurance because the sex difference in performance can be examined for each split discipline such as swimming, cycling and running and for the overall race time (Lepers and Maffiuletti 2011). Since the first Ironman distance triathlon held in Hawaii in 1978 consisting in 3.8 km swimming, 180 km cycling and 42.195 km running (Lepers 2008), the ultra-distance triathlon has been expanded to the Double Deca Iron ultra-triathlon distance consisting in 76 km swimming, 3,600 km cycling, and 840 km running (Lenherr et al. 2012).

Regarding the performance in the split disciplines in triathlon, recent studies showed that women were able to achieve a similar performance as men in ultra-distance swimming (Eichenberger et al. 2012a; 2012b). Indeed, a very recent study showed that women were able to outperform men in open-water ultra-distance swimming (Knechtle et al. 2014). In the 46 km ‘Manhattan Island Marathon Swim’ with water temperatures < 20°C, the top ten race times ever were significantly lower for women than for men. A potential explanation for the sex difference in ultra-endurance swimming performance could be anthropometric differences such as body fat between female and male ultra-endurance athletes. Female ultra-swimmers have a higher percent body fat of 30.7 ± 3.7% compared to male ultra-swimmers with 18.8 ± 4.5% (Weitkunat et al. 2012). Additionally, female swimmers had proportionately more fatty tissue caudally than male swimmers (McLean and Hinrichs 1998). These findings might explain why women were able to achieve men’s performance in ultra-swimming due to an improved buoyancy.

Regarding cycling as the second discipline in triathlon, little is known about the sex difference in ultra-cycling (Rüst et al. 2013; Shoak et al. 2013; Zingg et al. 2013). For shorter cycling distances, Schumacher et al. (2001) reported a sex difference of ~11% in all disciplines and at all ages when investigating results of the world track cycling championships in 200 m, 1,000 m, individual and team pursuit races for elite and junior athletes. For longer distances, women reduced the gender gap in the 720 km ‘Swiss Cycling Marathon’, the largest European qualifier for the ‘Race Across America’ (Zingg et al. 2013). In the ‘Race Across America’ itself, the sex difference remained, however, unchanged at ~19% for the annual fastest and at ~25% for the annual three fastest in the last 30 years (Rüst et al. 2013).

Analysing the sex difference in performance in long-distance triathlons could give some additional insights regarding the sex difference in ultra-swimming, ultra-cycling and ultra-running performances. The aim of this study was therefore to investigate the sex difference in ultra-triathlon performance for each split discipline and overall race time in different triathlon distances ranging from the Ironman distance (*i.e.* 3.8 km swimming, 180 km cycling and 42 km running) in ‘Ironman Hawaii’ to the Double Deca Iron ultra-triathlon distance (*i.e.* 76 km swimming, 3,600 km cycling and 840 km running) between 1978 and 2013. Based upon recent findings for single disciplines, we hypothesized that women would be able to reduce the gap with men in the swimming and running parts of ultra-triathlons.

## Methods

### Ethics

All procedures used in the study were approved by the Institutional Review Board of Kanton St. Gallen, Switzerland with a waiver of the requirement for informed consent of the participants given the fact that the study involved the analysis of publicly available data.

### Data sampling and data analysis

The data for this study was obtained from the race website of ‘Ironman World Championship’ (http://www.ironmanworldchampionship.com) for race results from ‘Ironman Hawaii’. For the triathlon distances between Double Iron and Double Deca Iron ultra-triathlon, the authors collected all race results from the website of the International Ultra-Triathlon Association (IUTA), http://www.iutasport.com) and from the race directors for all data not recorded on the website. Since the Double Iron ultra-triathlon held in Huntsville in 1994 included a downstream swimming split leading to unrealistic fast swimming split times, this specific race had to be excluded from analysis. Split times in swimming, cycling and running and overall race times of all finishers from ‘Ironman Hawaii’ and all ultra-triathlons up to Double Deca Iron ultra-triathlon were analysed. Due to the low number of female finishers in the longer ultra-triathlon distances (*i.e.* longer than the Triple Iron ultra-triathlon distance), we restricted our analysis to the three fastest triathletes ever and the annual three fastest. To find sex differences in the performance of men and women depending on the race distance, the top three men and women ever (*i.e.* the athletes with the three fastest overall race times ever) were sorted by race distance. From these athletes, the sex difference in performance was calculated using the equation ([time in women (min)]–[time in men (min)]/[time in men (min)] × 100), where the sex difference was calculated for every pair of equally placed athletes (*i.e.* between male and female winner, between male and female 2nd place, etc*.*) before calculating mean value and standard deviation of all the pairings. In order to facilitate reading all sex differences were transformed to absolute values before analysing. The sex difference between the annual top three men and women (*i.e.* annual three fastest overall race times) were determined and analysed regarding the change in sex difference over time.

### Statistical analysis

Each data set was tested for normal distribution and for homogeneity of variances prior to statistical analyses. Normal distribution was tested using a D’Agostino and Pearson omnibus normality test and homogeneity of variances was tested using a Levene’s test. Subsequent statistical tests were corrected according to these results if indicated and necessary. Single and multi-level regression analyses were used to investigate changes in speed and sex difference. A hierarchical regression model was used to avoid the impact of a cluster-effect on results in case one athlete finished more than once in the annual top three. Since the change in sex difference in endurance is assumed to be non-linear (Reinboud 2004), we calculated both the linear and the non-linear regression model for performance and sex difference in swimming, cycling, running and overall race time. In total, over 90 different models were tested in order to find the model that fits the data the best. Among these models, polynomial regression from degree 2 to 20, exponential models, growth models, sigmoidal models, decline models and others were included. In order to find the best model explaining the trend of the data, we compared the best-fit non-linear models to the linear model using AIC (Akaike’s Information Criteria) and F-test. Since the tested sets of data showed a normal distribution as well as homogeneity of variances, the performance between two groups (*e.g.* men *versus* women), was compared using a Student’s *t*-test. Additionally, a potential relationship between the sex difference of the three fastest competitors and total race time in men was investigated using Pearson’s correlation analysis. Statistical analyses were performed using IBM SPSS Statistics (Version 22, IBM SPSS, Chicago, IL, USA) and GraphPad Prism (Version 6.01, GraphPad Software, La Jolla, CA, USA). Significance was accepted at *P* < 0.05 (two-sided for *t*-tests). Data in the text and figures are given as mean ± standard deviation (SD).

## Results

### Participation trends

Between 1978 and 2013, a total of 46,123 athletes (*i.e.* 9,802 women and 46,123 men) finished ‘Ironman Hawaii’ and ultra-triathlons longer than the ‘Ironman Hawaii’ (Table 1). Women accounted on average for 11.9 ± 5.8% of the total field (Table 1). The percentage of female participation was highest in ‘Ironman Hawaii’ and lowest in Deca Iron ultra-triathlon (Table 1).

**Table 1:** **Number of female and male finishers in all races**

Race	Female finishers	Male finishers	Overall finishers	Women (%)	Men (%)
Ironman Hawaii (1978–2013)	9,502	33,459	42,961	22.1	77.9
Double Iron ultra-triathlon (1985–2013)	179	1,731	1,910	10.3	89.7
Triple Iron ultra-triathlon (1988–2013)	98	918	1,016	10.7	89.3
Quadruple Iron ultra-triathlon (1989–2013)	3	39	42	7.1	92.9
Quintuple Iron ultra-triathlon (1991–2013)	13	59	72	18.0	82.0
Deca Iron ultra-triathlon (1992–2013)	8	123	128	6.5	93.5
Double Deca Iron ultra-triathlon (1998–2013)	1	10	11	9.1	90.1

### Changes in performance across years

**Figure 1:**
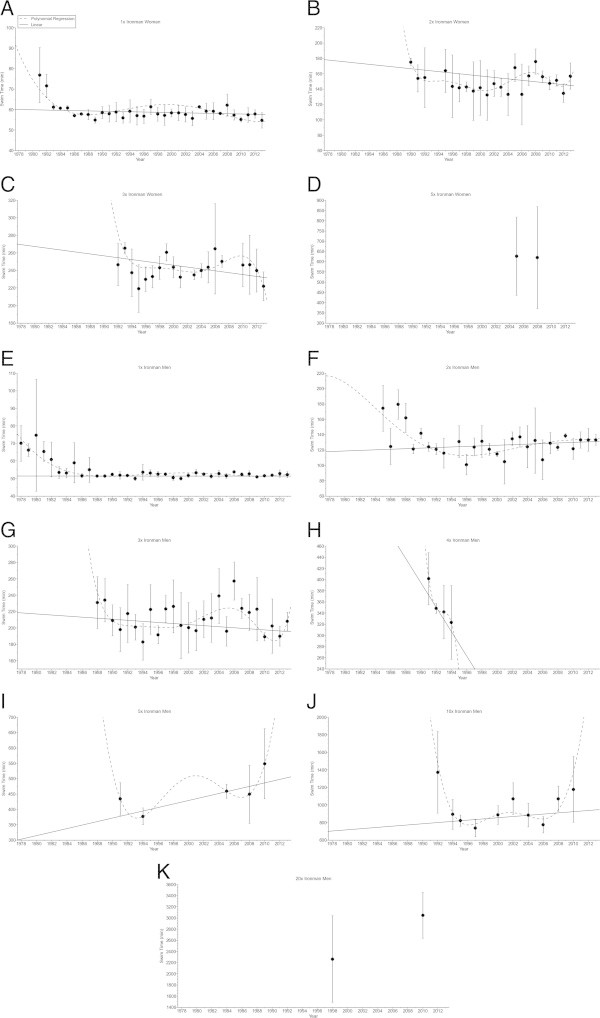
**Swimming split times for the annual three fastest women and men from Ironman to Double Deca Iron ultra-triathlon for Ironman women (Panel A), Double Iron women (Panel B), Triple Iron women (Panel C), Quintuple Iron women (Panel D), Ironman men (Panel E), Double Iron men (Panel F), Triple Iron men (Panel G), Quadruple Iron men (Panel H), Quintuple Iron men (Panel I), Deca Iron men (Panel J), Double Deca Iron men (Panel K).**

**Figure 2:**
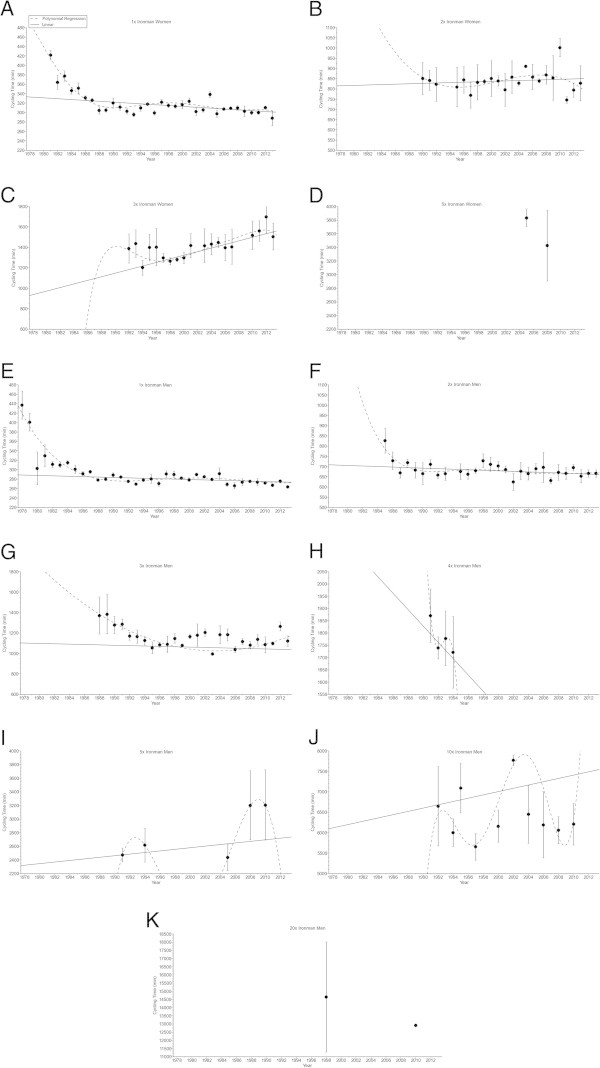
**Cycling split times for the annual three fastest women and men from Ironman to Double Deca Iron ultra-triathlon for Ironman women (Panel A), Double Iron women (Panel B), Triple Iron women (Panel C), Quintuple Iron women (Panel D), Ironman men (Panel E), Double Iron men (Panel F), Triple Iron men (Panel G), Quadruple Iron men (Panel H), Quintuple Iron men (Panel I), Deca Iron men (Panel J), Double Deca Iron men (Panel K).**

**Figure 3:**
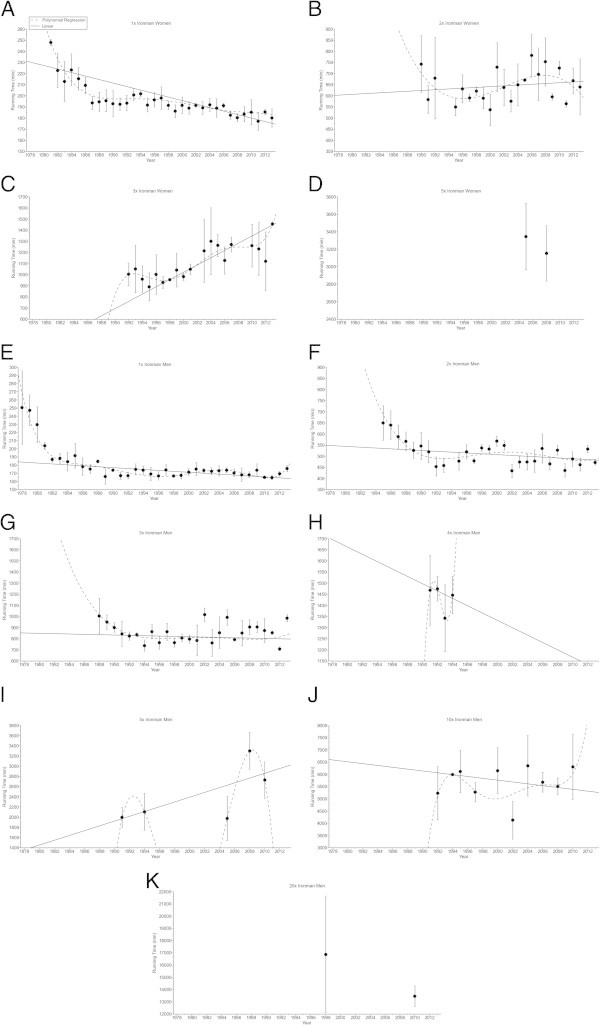
**Running split times for the annual three fastest women and men from Ironman to Double Deca Iron ultra-triathlon for Ironman women (Panel A), Double Iron women (Panel B), Triple Iron women (Panel C), Quintuple Iron women (Panel D), Ironman men (Panel E), Double Iron men (Panel F), Triple Iron men (Panel G), Quadruple Iron men (Panel H), Quintuple Iron men (Panel I), Deca Iron men (Panel J), Double Deca Iron men (Panel K).**

**Figure 4:**
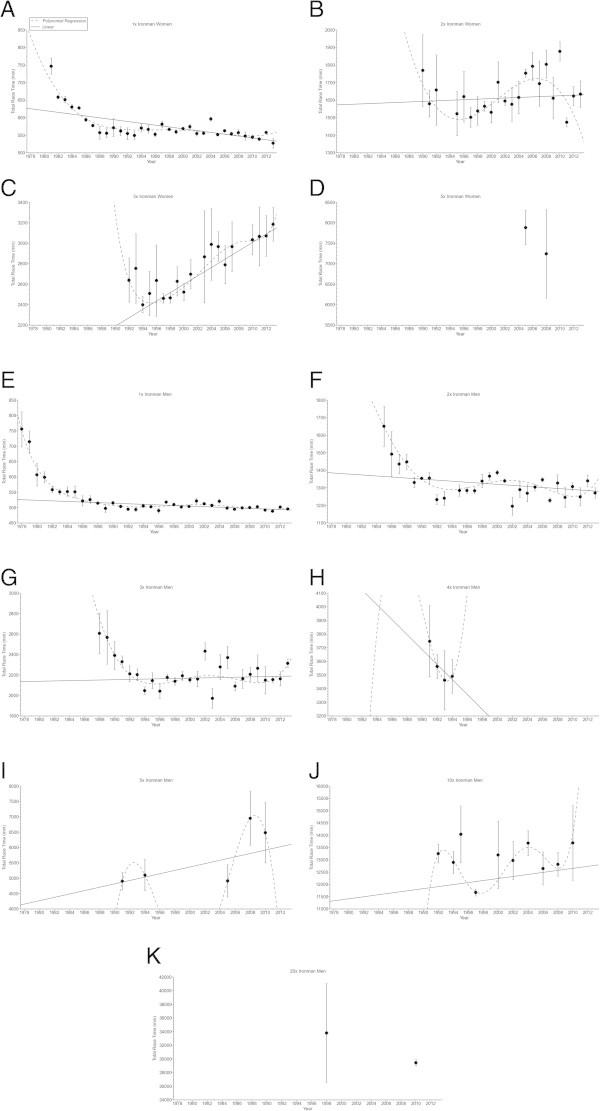
**Overall race times for the annual three fastest women and men from Ironman to Double Deca Iron ultra-triathlon for Ironman women (Panel A), Double Iron women (Panel B), Triple Iron women (Panel C), Quintuple Iron women (Panel D), Ironman men (Panel E), Double Iron men (Panel F), Triple Iron men (Panel G), Quadruple Iron men (Panel H), Quintuple Iron men (Panel I), Deca Iron men (Panel J), Double Deca Iron men (Panel K).**

In ‘Ironman Hawaii’, swimming split times (Figure 1) decreased non-linearly (*i.e.* polynomial regression 4th degree) (Table 2) in both women and men. From Double Iron to Deca Iron ultra-triathlon, no changes in swimming split times were found (Table 2). Cycling split times (Figure 2) decreased non-linearly in male (*i.e.* polynomial regression 4th degree) and female (*i.e.* polynomial regression 13th degree) Ironman triathletes (Table 3), and in male Double (*i.e.* polynomial regression 4th degree) and male Triple Iron ultra-triathletes (*i.e.* polynomial regression 2nd degree) (Table 3). In Quintuple Iron ultra-triathlon, however, cycling split times increased non-linearly in men (*i.e.* polynomial regression 4th degree) (Table 3). No changes were found for the other distances (Table 3). Running split times (Figure 3) decreased non-linearly in male (*i.e.* polynomial regression 8th degree) and female (*i.e.* polynomial regression 5th degree) Ironman triathletes (Table 4). In distances longer than the Ironman, running split times decreased non-linearly in male Double Iron ultra-triathletes (*i.e.* polynomial regression 4th degree) (Table 4). In Triple Iron ultra-triathlon, running split times increased non-linearly in women (*i.e.* polynomial regression 5th degree) (Table 4). In Quintuple Iron ultra-triathlon, running split times increased non-linearly in men (*i.e.* polynomial regression 4th degree) (Table 4). Overall race times (Figure 4) decreased non-linearly in male (*i.e.* polynomial regression 18th degree) and female (*i.e.* polynomial regression 4th degree) Ironman triathletes (Table 5). In distances longer than the Ironman, overall race times decreased non-linearly in male (*i.e.* polynomial regression 5th degree) Double and male (*i.e.* polynomial regression 5th degree) Triple Iron ultra-triathletes (Table 5). In Triple and Quintuple Iron ultra-triathlon, however, race times increased linearly across years in women and non-linearly in men (*i.e.* polynomial regression 4th degree) (Table 5).

**Table 2:** **Multi-level regression analyses for changes in swimming split times across years with correction for multiple finishes for the annual three fastest women and men**

Distance	Sex	***ß***	SE (***ß***)	Stand. ***ß***	T	***P***
**Ironman**	**Female**	−0.203	0.054	−0.358	−3.777	< 0.001
**Double Iron**	**Female**	−0.215	0.429	−0.062	−0.500	0.619
**Triple Iron**	**Female**	−0.042	0.442	−0.013	−0.096	0.924
**Quintuple Iron**	**Female**	−2.222	60.349	−0.018	−0.037	0.972
**Ironman**	**Male**	−0.338	0.065	−0.451	−5.196	< 0.001
**Double Iron**	**Male**	−0.525	0.321	−0.175	−1.635	0.106
**Triple Iron**	**Male**	−0.180	0.417	−0.049	−0.431	0.667
**Quadruple Iron**	**Male**	−24.133	11.354	−0.558	−2.126	0.059
**Quintuple Iron**	**Male**	5.382	2.526	0.509	2.131	0.053
**Deca Iron**	**Male**	0.727	8.419	0.016	0.086	0.932

**Table 3:** **Multi-level regression analyses for changes in cycling split times across years with correction for multiple finishes for the annual three fastest women and men**

Distance	Sex	***ß***	SE (***ß***)	Stand. ***ß***	T	***P***
**Ironman**	**Female**	−1.816	0.226	−0.632	−8.026	< 0.001
**Double Iron**	**Female**	0.985	1.320	0.093	0.746	0.458
**Triple Iron**	**Female**	12.086	2.604	0.530	4.641	< 0.001
**Quintuple Iron**	**Female**	−135.778	102.897	−0.551	−1.320	0.257
**Ironman**	**Male**	−2.167	0.255	−0.636	−8.495	< 0.001
**Double Iron**	**Male**	−1.960	0.560	−0.355	−3.497	0.001
**Triple Iron**	**Male**	−6.017	1.563	−0.404	−3.849	< 0.001
**Quadruple Iron**	**Male**	−40.933	27.098	−0.431	−1.511	0.162
**Quintuple Iron**	**Male**	32.680	13.618	0.554	2.400	0.032
**Deca Iron**	**Male**	−11.336	23.739	−0.090	−0.478	0.637

**Table 4:** **Multi-level regression analyses for changes in running split times across years with correction for multiple finishes for the annual three fastest women and men**

Distance	Sex	***ß***	SE (***ß***)	Stand. ***ß***	T	***P***
**Ironman**	**Female**	−1.228	0.115	−0.734	−10.647	< 0.001
**Double Iron**	**Female**	1.744	1.768	0.122	0.986	0.328
**Triple Iron**	**Female**	18.694	3.338	0.603	5.600	< 0.001
**Quintuple Iron**	**Female**	−64.111	95.465	−0.318	−0.672	0.539
**Ironman**	**Male**	−1.310	0.167	−0.606	−7.834	< 0.001
**Double Iron**	**Male**	−3.673	0.709	−0.490	−5.182	< 0.001
**Triple Iron**	**Male**	−0.261	1.600	−0.019	−0.163	0.871
**Quadruple Iron**	**Male**	−19.733	30.924	−0.198	−0.638	0.538
**Quintuple Iron**	**Male**	46.406	17.218	0.599	2.695	0.018
**Deca Iron**	**Male**	15.199	30.200	0.095	0.503	0.619

**Table 5:** **Multi-level regression analyses for change in overall race times cross years with correction for multiple finishes for the annual three fastest women and men**

Distance	Sex	***ß***	SE (***ß***)	Stand. ***ß***	T	***P***
**Ironman**	**Female**	−3.031	0.345	−0.665	−8.781	< 0.001
**Double Iron**	**Female**	3.404	2.436	0.172	1.398	0.167
**Triple Iron**	**Female**	31.511	4.612	0.678	6.833	< 0.001
**Quintuple Iron**	**Female**	−214.111	223.258	−0.432	−0.959	0.392
**Ironman**	**Male**	−3.646	0.419	−0.645	−8.696	< 0.001
**Double Iron**	**Male**	−5.999	1.110	−0.506	−5.406	< 0.001
**Triple Iron**	**Male**	−6.678	2.479	−0.295	−2.694	0.009
**Quadruple Iron**	**Male**	−87.100	45.689	−0.516	−1.906	0.086
**Quintuple Iron**	**Male**	84.992	29.276	0.627	2.903	0.012
**Deca Iron**	**Male**	7.978	30.303	0.050	0.263	0.794

### Changes in sex difference across years

**Figure 5:**
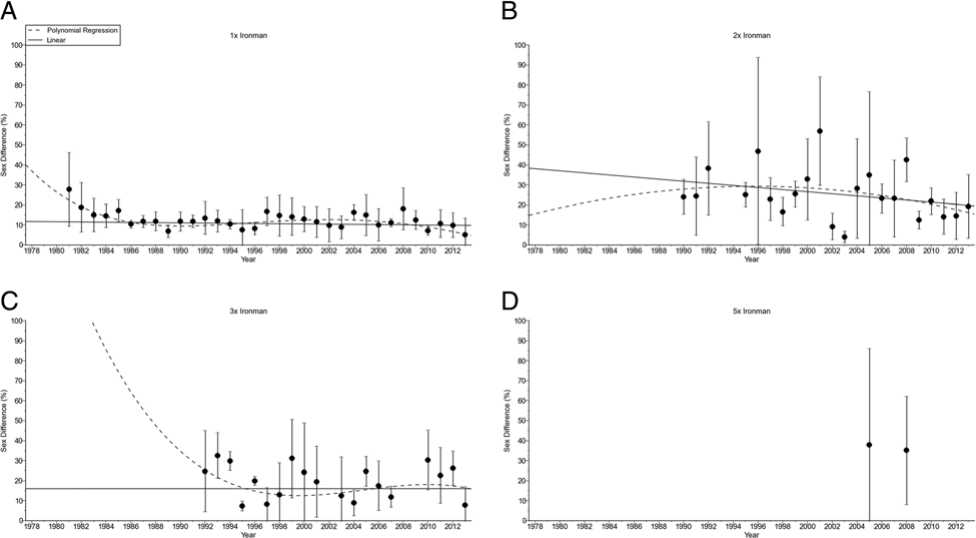
**Sex difference in swimming for the annual three fastest women and men from Ironman to Double Deca Iron ultra-triathlon for Ironman (Panel A), Double Iron (Panel B), Triple Iron (Panel C) and Quintuple Iron (Panel D).**

**Figure 6:**
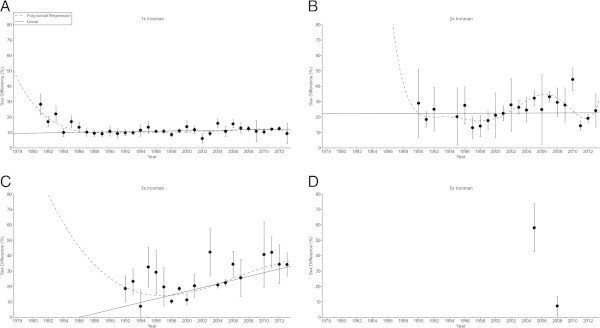
**Sex difference in cycling for the annual three fastest women and men from Ironman to Double Deca Iron ultra-triathlon for Ironman (Panel A), Double Iron (Panel B), Triple Iron (Panel C) and Quintuple Iron (Panel D).**

**Figure 7:**
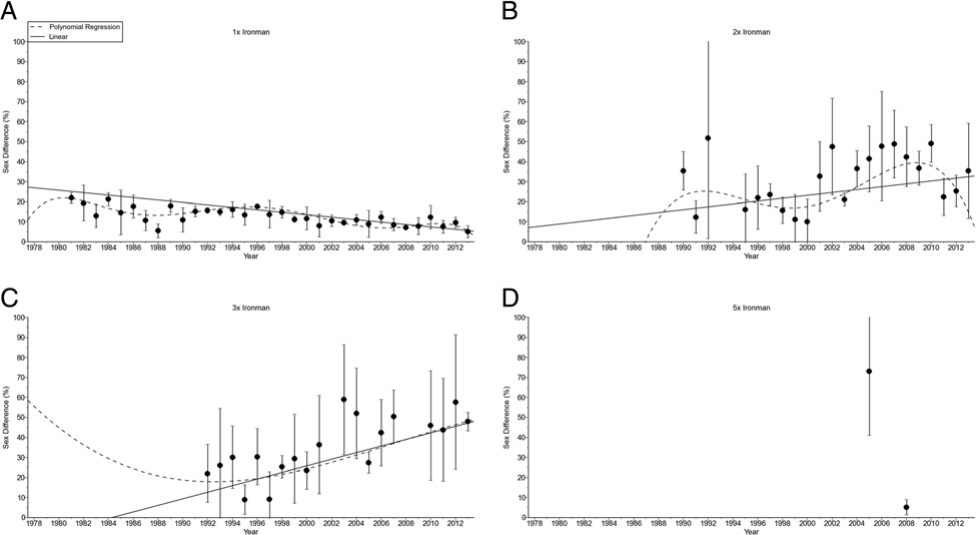
**Sex difference in running for the annual three fastest women and men from Ironman to Double Deca Iron ultra-triathlon for Ironman (Panel A), Double Iron (Panel B), Triple Iron (Panel C) and Quintuple Iron (Panel D).**

**Figure 8:**
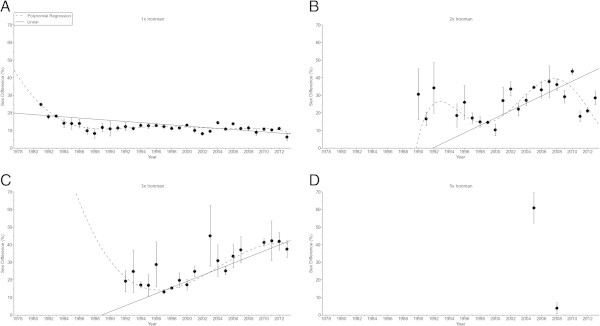
**Sex difference in overall race time for the annual three fastest women and men from Ironman to Double Deca Iron ultra-triathlon for Ironman (Panel A), Double Iron (Panel B), Triple Iron (Panel C) and Quintuple Iron (Panel D).**

The sex difference in the swimming split time (Figure 5) decreased non-linearly (Table 6) in ‘Ironman Hawaii’ (*i.e.* polynomial regression 5th degree). For the other distances, no changes were found (Table 6). In cycling (Figure 6), the sex difference decreased non-linearly (Table 7) in ‘Ironman Hawaii’ (*i.e.* polynomial regression 5th degree) but increased non-linearly in Triple Iron ultra-triathletes (*i.e.* polynomial regression 3rd degree) (Table 7). The sex difference in the running split time (Figure 7) decreased non-linearly (Table 8) in ‘Ironman Hawaii’ (*i.e.* polynomial regression 9th degree). In Triple Iron ultra-triathlon, the sex difference increased linearly (Table 8). For overall race time (Figure 8), the sex difference decreased non-linearly (Table 9) in ‘Ironman Hawaii’ (*i.e.* polynomial regression 8th degree). In Double Iron ultra-triathlon, the sex difference in overall race time increased non-linearly (*i.e.* polynomial regression 5th degree) (Table 9). In Triple Iron ultra-triathlon, the sex difference increased non-linearly (*i.e.* polynomial regression 3rd degree) (Table 9).

**Table 6:** **Multi-level regression analyses for change in sex difference in swimming across years with correction for multiple finishes**

Distance	***ß***	SE (***ß***)	Stand. ***ß***	T	***P***
**Ironman**	−0.187	0.077	−0.239	−2.428	0.017
**Double Iron**	−0.868	0.487	−0.217	−1.782	0.079
**Triple Iron**	−0.169	0.285	−0.080	−0.593	0.556
**Quintuple Iron**	−0.884	10.660	−0.041	−0.083	0.938

**Table 7:** **Multi-level regression analyses for change in sex difference in cycling across years with correction for multiple finishes**

Distance	***ß***	SE (***ß***)	Stand. ***ß***	T	***P***
**Ironman**	−0.155	0.050	−0.299	−3.089	0.003
**Double Iron**	0.277	0.214	0.160	1.294	0.200
**Triple Iron**	0.992	0.245	0.479	4.051	< 0.001
**Quintuple Iron**	−16.983	3.264	−0.933	−5.204	0.007

**Table 8:** **Multi-level regression analyses for change in sex difference in running across years with correction for multiple finishes**

Distance	***ß***	SE (***ß***)	Stand. ***ß***	T	***P***
**Ironman**	−0.316	0.052	−0.527	−6.114	< 0.001
**Double Iron**	0.651	0.356	0.223	1.830	0.072
**Triple Iron**	1.668	0.385	0.504	4.332	< 0.001
**Quintuple Iron**	−22.678	6.222	−0.877	−3.645	0.022

**Table 9:** **Multi-level regression analyses for change in sex difference in overall race time across years with correction for multiple finishes**

Distance	***ß***	SE (***ß***)	Stand. ***ß***	T	***P***
**Ironman**	−0.200	0.032	−0.538	−6.279	< 0.001
**Double Iron**	0.411	0.179	0.276	2.295	0.025
**Triple Iron**	1.254	0.181	0.683	6.943	< 0.001
**Quintuple Iron**	−19.022	1.769	−0.983	−10.755	< 0.001

### Performance and sex difference of the fastest finishers ever

**Figure 9:**
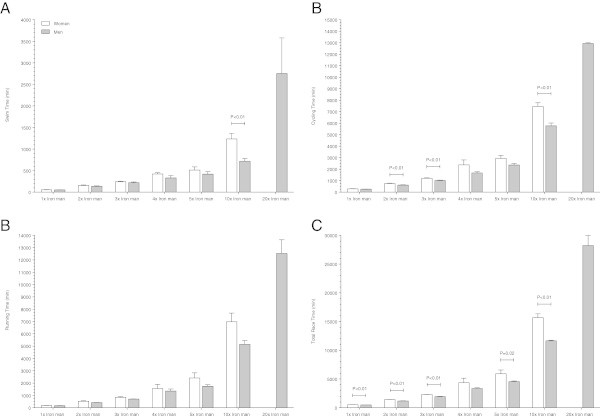
**Split and overall race times of the three fastest women and men ever for swimming (Panel A), cycling (Panel B), running (Panel C) and overall race time (Panel D).**

**Figure 10:**
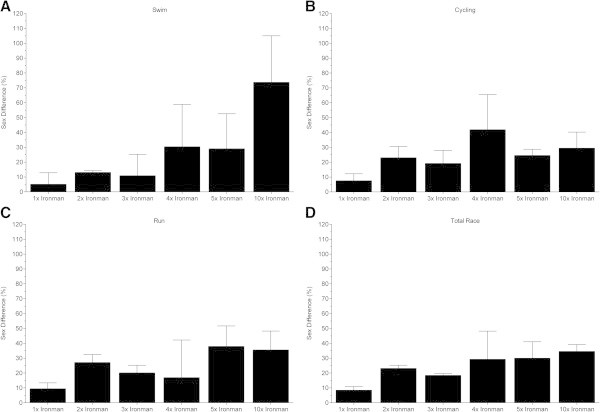
**Sex difference in split and overall race times of the three fastest women and men ever for swimming (Panel A), cycling (Panel B), running (Panel C) and overall race time (Panel D).**

**Figure 11:**
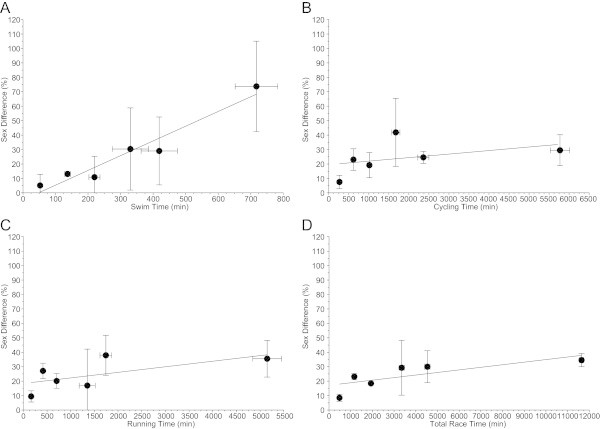
**Sex difference in relation to split and overall race times for the three fastest competitors ever across all distances for swimming (Panel A), cycling (Panel B), running (Panel C) and overall race time (Panel D).**

When the fastest three women and men ever were considered (Figure 9), men were faster than women in both the split times (Panels A-C) and overall race times (Panel D). Figure 10 presents the sex difference for the three fastest finishers ever with split (Panels A-C) and overall race times (Panel D). Men were always faster than women. For the three fastest finishers ever, the sex differences in swimming were for Ironman, Double, Triple, Quadruple, Quintuple and Deca Iron ultra-triathlon 5.2 ± 7.7%, 13.1 ± 1.2%, 10.9 ± 14.4%, 30.4 ± 28.5%, 29.0 ± 23.5%, and 73.7 ± 31.3%, respectively. In cycling, the sex differences were 7.6 ± 4.6%, 23.1 ± 7.5%, 19.2 ± 8.7%, 41.9 ± 23.5%, 24.6 ± 4.1%, and 29.5 ± 10.7%, respectively. In running, the values were 9.5 ± 3.9%, 27.1 ± 5.3%, 20.1 ± 5.1%, 16.9 ± 25.3%, 37.9 ± 13.8%, and 35.5 ± 12.7%, respectively. For overall race times, the sex differences were 8.5 ± 2.5%, 23.1 ± 2.2%, 18.4 ± 1.2%, 29.3 ± 19.0%, 30.0 ± 11.0%, and 34.6 ± 4.6%, respectively. The mean sex differences for swimming, cycling, running and overall race times for all distances from Ironman to Deca Iron ultra-triathlon were 27.0 ± 17.8%, 24.3 ± 9.9%, 24.5 ± 11.0%, and 24.0 ± 6.7%, respectively. Figure 11 presents the relationship between sex difference and performance for split times (Panels A-C) and overall race times (Panel D) for the three fastest finishers ever. The sex difference increased with increasing race distance in swimming (r^2^ = 0.93, *P* = 0.001), but not in cycling (r^2^ = 0.18, *P* = 0.38), running (r^2^ = 0.41, *P* = 0.16), and overall race time (r^2^ = 0.59, *P* = 0.07).

## Discussion

This study investigated the sex difference in performance for each discipline and overall race time for the fastest competitors in ultra-distance triathlons from the Ironman distance in ‘Ironman Hawaii’ to the Double Deca Iron ultra-triathlon distance between 1978 and 2013. It was hypothesized that women would be able to reduce the gap with men in both swimming and running parts in ultra-triathlons. The main findings were that (*i*) the fastest men were faster than the fastest women regarding overall race times, (*ii*) women reduced the sex difference in the shorter ultra-triathlon distances (*i.e.* Ironman distance) but extended the sex difference in longer distances (*i.e.* Double and Triple Iron ultra-triathlon) and (*iii*) in contrast to our hypothesis, the sex difference in the swimming split increased with increasing race distance.

### Low female participation in ultra-endurance triathlon

Female participation was highest in ‘Ironman Hawaii’ and lowest in Deca Iron ultra-triathlon. In Double Deca Iron ultra-triathlon, only one woman ever finished to date. The low number of women in the very long ultra-distance triathlons (*i.e.* longer than the Triple Iron ultra-triathlon) might considerably influence the sex difference in triathlon performance. The offer of Ironman races is considerably higher (http://www.ironman.com/triathlon/events) compared to races longer than the Ironman distance (http://www.iutasport.com). This large difference in offer might influence the participation. For running, Coast et al. (2004) compared the world best running performances at distances from 100 m to 200 km by the end of 2002. Men were faster than women with an average sex difference of ~12.4% and longer distances were associated with greater sex differences. The authors argued that their results might be confounded by the lower number of women competing in longer distance events. Generally, men are over-represented in sports (Deaner 2013) and the faster performance in men compared to women is most probably due to men’s greater training motivation (Deaner 2013). Popular modern male sports require the skills needed for success in male-male physical competition (Lombardo 2012) whereas as female ultra-endurance athletes are task-oriented, internally motivated, health, and financially conscious individuals (Krouse et al. 2011).

### Differences in motivation between female and male athletes

An explanation for the sex difference in both participation and performance in ultra-endurance could be a difference in the motivation between women and men to participate in an ultra-endurance race. Female participation in these ultra-triathlons was generally low at ~12%. Personality, motivation, and goal orientation have been investigated in endurance athletes such as runners (Frederick and Ryan 1993) and participants in different sports disciplines (Frederick and Ryan 1993; Gill and Overdorf 1994). Women were motivated to exercise regularly in order to reduce their body fat, to increase their physical fitness, or to improve their social interactions (Bond 2005; Frederick and Ryan 1993; Gill and Overdorf 1994; Hodge et al. 2008; Levy 2002). The aspect of competing and winning a race seemed to be of lower importance for women compared to men. For example, for female marathoners, the aspect of social affiliation and improving physical fitness is more important than achievement and personal accomplishment (Masters and Ogles 1995; Ogles and Masters 2003). Krouse et al. (2011) investigated the influence of motivation, goal orientation and training on performance for female ultra-marathoners. General health orientation and psychological coping were the strongest motivational factors for female ultra-marathoners.

### Sex difference and race distance

It was hypothesized that women would reduce the gap with men in both swimming and running parts of ultra-triathlons. Although women reduced the sex difference across years in the shorter ultra-triathlon distances such as the Ironman distance, they extended the sex difference in longer distances such as the Double and the Triple Iron ultra-triathlon distance. Furthermore, the sex difference remained unchanged at ~24-27% for split and overall race times when the fastest three performances ever for all distances from Ironman to Double Deca Iron ultra-triathlon were considered. Recent studies investigating ultra-endurance athletes competing in single disciplines showed that women reduced the gap to men in ultra-swimming (Rüst et al. 2014) and ultra-running (Zingg et al. 2014b). Although women were able to outperform men in ultra-swimming under certain conditions (*i.e.* very long distance and very cold water) (Knechtle et al. 2014), is seemed unlikely that women will outperform men in ultra-marathon running (Zingg et al. 2014b) and ultra-cycling (Rüst et al. 2013).

The sex difference in performance between female and male endurance athletes might be explained in part by differences in anthropometric characteristics such as skeletal muscle mass and body fat between female and male athletes. Knechtle et al. (2011a) argued that the increase in sex difference with increasing length of an ultra-endurance performance such as an ultra-triathlon was most probably due to the lower skeletal muscle mass in women compared to men. It has been shown that male ultra-endurance athletes had a higher skeletal muscle mass than female ultra-endurance athletes (Knechtle et al. 2010a; 2010b; 2011b; Weitkunat et al. 2012). Male Ironman triathletes with ~41 kg skeletal muscle mass had a ~46% higher skeletal muscle mass compared to female Ironman triathletes with ~28 kg of skeletal muscle mass (Knechtle et al. 2010a). For ultra-runners, male ultra-runners with ~38 kg skeletal muscle mass (Knechtle et al. 2011b) had a ~38% higher muscle mass compared to female ultra-runners with ~27.4 kg (Knechtle et al. 2010b). For ultra-swimmers, the sex difference in skeletal muscle mass was considerably higher compared to runners (Weitkunat et al. 2012). Male open-water ultra-swimmers with ~42 kg skeletal muscle mass had a ~45% more skeletal muscle mass compared to female open-water ultra-swimmers with ~29 kg skeletal muscle mass (Weitkunat et al. 2012). These differences in skeletal muscle mass between female and male ultra-endurance athletes might explain the increase in sex difference with increasing length of an endurance performance.

Another major predictor variable for a successful outcome in endurance competitions is percent body fat. It has been shown that lower body fat was associated with faster race times in men (Knechtle et al. 2011c; Rüst et al. 2012). Mean body fat percentages for male and female Ironman triathletes were ~14% and ~23%, respectively (Knechtle et al. 2010a). The higher skeletal muscle mass and the lower body fat percentage in men may support the theory of a biological based performance difference of female and male ultra-endurance athletes.

A further aspect is that performance in cycling is related to leg power (Hopker et al. 2010). An ultra-endurance performance leads to a decrease in lean body mass (Schütz et al. 2013). Since women have a lower skeletal muscle mass, a prolonged cycling performance might lead to a higher relative decrease in lower leg skeletal muscle mass in women compared to men and therefore, cycling performance becomes more reduced in women compared to men.

### Women extended the sex difference in swimming with increasing race distance

It was hypothesized that women would be able to achieve better swimming performances than men but in contrast to our hypothesis, the sex difference in the swimming split increased with increasing race distance in these ultra-triathletes. A potential explanation could be the ‘history’ of the athletes. Triathletes have to compete in three different disciplines and the background might be different for each athlete. Since cycling and running performance are predictive for ultra-triathlon performance (Knechtle et al. 2007; Knechtle and Kohler 2009), the background of an ultra-triathlete might rather be running or cycling than swimming. For elite open-water ultra-swimmers, however, women improved in long-distance swimming in recent years (Eichenberger et al. 2012b; Vogt et al. 2013; Zingg et al. 2014a). The sex difference in the world’s best 10 km open-water swimmers is only ~7% (Vogt et al. 2013). In open-water swimming, women are even able to achieve men’s performance (Eichenberger et al. 2012b). However, when the the changes in swimming speeds and sex differences for elite male and female swimmers competing in 5 km, 10 km and 25 km open-water FINA World Cup races held between 2000 and 2012 were investigated (Zingg et al. 2014c), elite female open-water ultra-distance swimmers improved in 10 km but impaired in 25 km leading to a linear decrease in sex difference in 10 km and a linear increase in sex difference in 25 km. The linear changes in sex differences suggested that women would improve in the near future in 10 km, but not in 25 km (Zingg et al. 2014c).

## Conclusions

Although women reduced the sex difference in the shorter ultra-triathlon distances (*i.e.* Ironman distance), the sex difference in longer triathlon distances (*i.e.* Double and the Triple Iron ultra-triathlon distance) remained greater compared to the single Ironman distance. The sex difference for split times and overall race times remained unchanged at ~24-27% for the fastest three performances ever for all distances. Additionally, the sex difference in the swimming split increased significantly with increasing race distance. The most likely reasons for the sex difference in ultra-triathlon performance are the lower number in female participation in these races, the lower muscle mass in women compared to men and the different motivational trends in women compared to men. It seems very unlikely that women will ever outrun men in ultra-distance triathlons from the Ironman to the Double Deca Iron ultra-triathlon distance.
